# From rough to precise: PD-L1 evaluation for predicting the efficacy of PD-1/PD-L1 blockades

**DOI:** 10.3389/fimmu.2022.920021

**Published:** 2022-08-03

**Authors:** Xuan Zhao, Yulin Bao, Bi Meng, Zijian Xu, Sijin Li, Xu Wang, Rui Hou, Wen Ma, Dan Liu, Junnian Zheng, Ming Shi

**Affiliations:** ^1^ Jiangsu Center for the Collaboration and Innovation of Cancer Biotherapy, Cancer Institute, Xuzhou Medical University, Xuzhou, China; ^2^ College of Pharmacy, Xuzhou Medical University, Xuzhou, China

**Keywords:** PD-L1, immune checkpoint inhibitors, tumor immunotherapy, biomarker, liquid biopsy, tumor microenvironment

## Abstract

Developing biomarkers for accurately predicting the efficacy of immune checkpoint inhibitor (ICI) therapies is conducive to avoiding unwanted side effects and economic burden. At the moment, the quantification of programmed cell death ligand 1 (PD-L1) in tumor tissues is clinically used as one of the combined diagnostic assays of response to anti-PD-1/PD-L1 therapy. However, the current assays for evaluating PD-L1 remain imperfect. Recent studies are promoting the methodologies of PD-L1 evaluation from rough to precise. Standardization of PD-L1 immunohistochemistry tests is being promoted by using optimized reagents, platforms, and cutoff values. Combining novel *in vivo* probes with PET or SPECT will probably be of benefit to map the spatio-temporal heterogeneity of PD-L1 expression. The dynamic change of PD-L1 in the circulatory system can also be realized by liquid biopsy. Consider PD-L1 expressed on non-tumor (immune and non-immune) cells, and optimized combination detection indexes are further improving the accuracy of PD-L1 in predicting the efficacy of ICIs. The combinations of artificial intelligence with novel technologies are conducive to the intelligence of PD-L1 as a predictive biomarker. In this review, we will provide an overview of the recent progress in this rapidly growing area and discuss the clinical and technical challenges.

## Introduction

The immune checkpoint inhibitors (ICIs) targeting PD-1 and PD-L1, which have improved the outcomes of patients with various forms of advance-stage tumor, mark a landmark breakthrough in cancer treatment (1). Various anti-PD-1/PD-L1 drugs are currently clinically approved. At the moment, owing to the low objective remission rate (2), reliable biomarkers for screening patients eligible for anti-PD-1/PD-L1 therapies are urgently required.

To a certain degree, PD-L1 is considered as a good predictor of the response to ICI treatment in clinical cancer immunotherapy. Some reports suggest that high PD-L1 expression in tumor tissues is positively correlated with the prognosis of patients receiving anti-PD-1/PD-L1 therapy (3, 4). The United States Food and Drug Administration (FDA) has approved immunohistochemistry (IHC) for assessing PD-L1 expression in patients with non-small cell lung cancer (NSCLC), melanoma, bladder cancer, and cervical cancer (5, 6). However, some clinical studies report that there is an insufficient direct correlation between PD-L1 expression and therapeutic impression (7). The effect of anti-PD-1/PD-L1 therapy is not only limited to patients with high PD-L1 expression but also to patients with low PD-L1 expression who can benefit from anti-PD-1/PD-L1 treatment (8–11). Check-Mate 017 and Check-Mate 057 studies demonstrate that PD-L1 expression is positively associated with a greater overall survival (OS) profit from nivolumab in NSCLC patients with ≥50% PD-L1 expression; however, an effective response is still observed in patients with ≤1% PD-L1 expression (12).

Furthermore, various factors have been associated with the failure of PD-L1 in predicting the efficacy of ICI therapy. This review clarifies the possible reasons why it is difficult for PD-L1 to predict the efficacy of immunotherapy accurately and discusses the strategies involved in acquiring precise predictions of immunotherapy response assessment.

## Advanced IHC protocols for PD-L1 detection

IHC and flow cytometry have been clinically used to detect PD-L1 expression in cancer tissues. However, conventional IHC assay lacks accuracy and reliability as the staining of cytoplasmic proteins interferes with the estimation of cell membrane proteins. Additionally, flow cytometry can only be used for fresh tissue sample detection, thus limiting its application (13). Fortunately, diverse optimizations are available in testing reagents, platforms, and scoring methods for PD-L1 IHC assessment (14, 15).

### Detection reagents

Various antibodies for PD-L1 IHC assessment are available (**Table 1**). The Blueprint PD-L1 Immunohistochemistry Comparability Phase II Project has been carried out to assess the clinical use of five commercial PD-L1 IHC assays, including 22C3, 28-8, SP142, SP263, and 73-10 (not in phase I) clones. According to the PD-L1 detections in a mixture of lung cancer samples (39 adenocarcinomas, 26 squamous cell carcinomas, six poorly differentiated non-small cell carcinomas, and 10 small cell carcinomas), there was a comparable sensitivity between 22C3, 28-8, and SP263 assays, while the sensitivity of SP142 assay was lower. Compared to other anti-PD-L1 antibody clones, 73-10 assay showed greater sensitivity (16). However, different reports have different conclusions. Dako’s Autostainer Link 48 and 22C3 anti-PD-L1 antibody, an automatic diagnostic assay, have been reported to be optimized for high sensitivity and specificity in patients with NSCLC who were treated with pembrolizumab (17). Adam et al. performed IHC tests on 41 NSCLC surgical specimens with five anti-PD-L1 monoclonal antibodies (28-8, 22C3, E1L3N, SP142, and SP263) in seven centers, including Dako Autostainer Link 48 (three centers), Leica Bond (two centers), or Ventana BenchMark Ultra (two centers) platforms. The results demonstrated that SP263 achieved the highest concordance rate in all platforms (18).

**Table 1:** Food and Drug Administration (FDA) and European Medicines Agency (EMA)-approved immune checkpoint blockades.

	Name	Trade name	IHC diagnostic assays		
			Antibody clone	Platform	Clinical application of cancer therapy
PD-1 inhibitors	Nivolumab	Opdivo	28-8	Dako Autostainer Link 48	NSCLC, UC, and HNSCC
	Pembrolizumab	Keytruda	22C3	Dako Autostainer Link 48	NSCLC, GEJ adenocarcinoma, ESCC, cervical cancer, UC, TNBC, and HNSCC
	Cemiplimab	Libtayo	22C3	Dako Autostainer Link 48	NSCLC
PD-L1 inhibitors	Atezolizumab	Tecentriq	SP142	Ventana Benchmark Ultra	UC, NSCLC, and TNBC
	Durvalumab	Imfinzi	SP263	Ventana Benchmark Ultra	UC

Data comes from FDA (https://www.fda.gov/) and EMA (https://www.ema.europa.eu/en).

To promote the consistency of PD-L1 measurement results, the International Association of Lung Cancer Research initiated the Blueprint project to compare the two detection platforms (Dako Autostainer Link 48 and Ventana Benchmark Ultra) and four antibodies (22C3, 28-8, P263, and SP142). Tests reveal consistent staining in three antibodies—22C3, 28-8, and SP263—when detecting PD-L1 expression in tumor cells, with the positive rate of SP142 antibody lower than the rest (19–21). However, owing to the low penetration of 22C3, 28-8, and the detection platform Autostainer Link 48, their application in PD-L1 assays are limited (22). Furthermore, according to the anti-PD-L1 IHC evaluation on 150 paired histological and cytological smears of NSCLC patients, Costantino et al. found that clone SP263 showed higher accuracy than 28-8 and 22-C3 clones, with good cyto-histological agreement when the cutoff value is 50% (23). Therefore, anti-PD-L1 SP263 IHC detection of PD-L1 in solid tumor tissues is recommended and prospective.

### Establishment of PD-L1 detection platforms

Various institutes are currently carrying out laboratory development tests (LDTs) which measure PD-L1 expression by adjusting conditions such as tissue controls, thresholds on existing testing platforms (Dako, Ventana, or Leica), and trailing antibodies from other clones that conflict with the detection platforms. The diagnostic results of LDTs vary greatly, failing to obtain a clinically acceptable diagnostic sensitivity (21), which can be attributed to the use of specific and different clones by different laboratories. According to a Canadian 22C3 IHC LDT validation project, it was possible to exploit highly accurate 22C3 IHC LDT for both 1 and 50% tumor proportion score (TPS) in NSCLC across 20 Canadian pathology laboratories with different platforms (24). Moreover, 22C3 PD-L1 IHC was cross-validated to detect PD-L1 expression in 23 NSCLC specimens by Benchmark Ultra (eight centers) and Leica Bond (12 centers) platforms. In addition, five centers performed Ventana SP263 assay. The results showed that SP263 assay was well concordant with 22C3 on Benchmark Ultra (25). It suggests that the establishment of a reference standard and unified test platform protocol, which is based on diagnostic accuracy, contributes to improve the sensitivity and specificity of the clinical application of LDTs. The improvement and application of 22C3 IHC LDT and SP263 IHC LDT for PD-L1 detection are recommended.

### Optimal cutoff value for PD-L1 measurement

The cutoff value of PD-L1 positivity chosen by different clinical institutions varies, mainly consisting of 1, 5, 10, and 50% (23). A study in renal cell carcinoma suggests that the objective response rate of atezolizumab in patients with PD-L1-positive is 56% when the cutoff value is 5%, whereas the objective response rate of patients with PD-L1-negative is 25%, with a significant difference. However, when the cutoff value is 1%, the objective response rates of positive and negative patients are 50 and 55%, respectively, with no statistical significance (13). Keynote-024 set the cutoff value to 50% or greater and showed that pembrolizumab significantly improved the prognosis of patients with NSCLC and PD-L1 ≥50%, with OS extending to 30 months, compared with 14.2 months for patients receiving chemotherapy (26). Generally, a higher cutoff value indicates a positive correlation with more accurate prediction; however, it also indicates the reduced sensitivity of the test and increased false negatives, which might neglect some patients who could benefit from anti-PD-1/PD-L1 therapy. Therefore, a method to determine practicable cutoff values of PD-L1 positivity is required.

### PD-L1 combined positive score

Apart from tumor cells, PD-L1 is also widely expressed in various immune cells (ICs) and non-ICs, such as T cells (27, 28), macrophages (29, 30), B cells (31, 32), dendritic cells (33), neutrophils (34), and fibroblasts (35). PD-L1 in non-tumor cells is involved in regulating antitumor immunity, which affects the efficacy of anti-PD-1/PD-L1 therapy in patients by interacting with tumor cells.

Sometimes, classifying PD-L1 as negative or positive in tumor tissue *via* TPS may be inaccurate. PD-L1 combined positive score (CPS) is calculated as the total number of PD-L1-positive cells (tumor cells, lymphocytes, and macrophages) divided by the amount of surviving tumor cells in the whole section and multiplied by 100 (36). During anti-PD-1/PD-L1 therapy, taking into account both PD-L1 expression on tumor cells and non-tumor cells could contribute to improving the accuracy of PD-L1 as a biomarker (37–40). Paintal et al. evaluated the consistency of PD-L1 CPS value in 20 paired head and neck squamous cell carcinoma cases, which were separated into two groups: excisional group (excisional biopsy and resection specimen) and small biopsy group. When a PD-L1 CPS of 20 was the cutoff, the accordance between the two groups was 90% but reduced to 70% at a threshold of PD-L1 CPS ≥1. This indicates that a higher PD-L1 CPS value correlates with a higher PD-L1 expression, providing a more reliable predictive accuracy (41). The phase III KEYNOTE-181 study in advanced oesophageal cancer indicates that pembrolizumab, a monoclonal antibody of PD-1, can prolong OS in patients with PD-L1 CPS ≥10 (42). However, another research in metastatic triple-negative breast cancer (TNBC) shows that pembrolizumab monotherapy in patients with PD-L1 CPS ≥1 or PD-L1 CPS ≥10 did not improve OS compared with chemotherapy, whereas PD-L1 CPS of 20 or more indicates a higher PD-L1 expression and a longer median OS in patients undergoing pembrolizumab treatment, which was approximately 18% of the total study population (43). Hence, a higher value of PD-L1 CPS is beneficial in accurately selecting subpopulations undergoing pembrolizumab monotherapy.

## Characteristics of PD-L1 in tumor tissues

### Intra-tumoral heterogeneity of PD-L1 expression

As PD-L1 expression is coupled with considerable intra-tumoral heterogeneity, it is difficult to judge the true PD-L1 status accurately in whole primary tumor sections using a single core biopsy (44). In NSCLC, based on PD-L1 IHC 22C3 pharmDx assay performed by Hwang et al., intra-tumoral heterogeneity is related to an increase in the proportion of patients with high PD-L1 expression in metastatic site biopsies and might result in the misjudgment of PD-L1 status (45). It is conducive to reduce the misclassification of PD-L1 expression when the size of biopsy is not less than 8 mm^2^. Using tissue microarrays, Stovgaard *et al.* evaluated PD-L1 expression in the surgical specimens of 110 patients with TNBC. A total of 31% (*n* = 34) of the cases showed an inconsistent PD-L1 expression on tumor cells (TCs) among the four different tissue microarray cores (each core ≥100 TCs). Moreover, compared with the tumor borders, the central areas contained almost no ICs. The staining results showed that the cores obtained from the center were negative for PD-L1 expression, whereas those from the border, with abundant ICs, were positive (46). Alexander et al. assessed PD-L1 expression *via* IHC using the SP263 clone. After digital imaging using a new “squares method”, the intra-tumoral heterogeneity of PD-L1 in resected primary NSCLCs was demonstrated to be variable in pattern and extent, with 78% for small scale (mm²), 50% for medium scale (cm²), and 46% for large scale (between tumor blocks) (47). Studies regarding thymoma and thymic carcinoma also report that the PD-L1 staining of different sections of the same tumor specimen from one patient differs (48). Therefore, the intra-tumoral biopsy location, core numbers, cell numbers, and sampling area significantly affect the determination of PD-L1 expression.

### Inter-tumoral (primary tumor *vs*. metastatic lesions) heterogeneity of PD-L1 expression

Numerous studies have reported that PD-L1 expression is highly heterogeneous between primary and metastatic tumor lesions. Therefore, ignoring the differences in PD-L1 expression between primary and metastatic sites could increase tumor misclassification risks and poor treatment decisions (45, 49). Tretiakova >et al. performed IHC assays to detect PD-L1 levels in 235 urothelial cancer tissue samples. The PD-L1 positivity rates differed between primary and metastatic sites, with primary tumor-positive rate of 28.9% (41/142), metastatic lymph node-positive rate of 16.9% (13/77), and positivity rate in distant metastatic tissues of 12.5% (2/16) (50). Another study noted that the proportion of primary tumor specimens (*n* = 1,285) with high PD-L1 expression is lower than that of metastatic specimens (n = 428). The proportion of primary tumor specimens with TPS ≥50% was 26.9%, while that of metastases was 38.3%, with a significant difference in lung cancer (45). Patients with metastatic NSCLC (*n* = 398) were divided into the lung, lymph node, and distant metastasis groups. In the lung and distant metastasis groups, higher PD-L1 levels corresponded with a better prediction of ICI therapy response rates and longer progression-free survival and OS. However, in the lymph node group, the PD-L1 levels were not significantly related to ICI therapy response rates (51).

### Dynamic expression of PD-L1 before and after the therapy

The dynamic expression of PD-L1 is not only regulated by cellular intrinsic factors (52) but also affected by disease progression and treatment schedules (53). Studies have found that PD-L1 expression changes before and after surgery (54), neoadjuvant chemotherapy, targeted therapy, or immunotherapy. In NSCLC, Hwang et al. identified that there was a 62% concordance in PD-L1 expression of biopsy–resection pairs (45). Matsumoto et al. (55) evaluated PD-L1 expression in puncture and surgical samples from 94 patients with pancreatic cancer. When the cutoff value was set to 5%, seven puncture and 16 surgical samples were PD-L1-positive, and the consistency rate was 44%. When the cutoff value was set to 10%, six puncture samples and 11 surgical samples were PD-L1 positive, with a consistency rate of 55%. Thus, the expression of PD-L1 is different before and after surgery. Another study using samples from patients with ovarian epithelial cancer undergoing neoadjuvant chemotherapy reported that 30% (15/50) were PD-L1-positive before treatment (cutoff value of PD-L1 is 5%), while 53% (27/51) were PD-L1-positive after treatment (56). Additionally, among the 13 patients with NSCLC treated with epidermal growth factor receptor tyrosine kinase inhibitor, Omori et al. report that PD-L1 expression in the tumor tissues increased significantly in five patients (57). Herbst et al. obtained biopsy samples from patients receiving atezolizumab treatment at different timepoints and proved that PD-L1 expression increases when the tumor volume decreases (58). Hence, PD-L1 detection is greatly influenced by the biopsy time and therapeutic regime. Moreover, continuous sampling at multiple timepoints could be used to optimize the current testing methods.

Therefore, the heterogeneous expression of PD-L1 in tumor tissues and the dynamic changes limit the feasibility of PD-L1-related IHC analysis in tissue biopsy and the accuracy of PD-L1 as a predictor of treatment efficacy (59). Ongoing studies have improved PD-L1 detection capability, with specimens being collected from a single core in one tumor section to multiple regions in different tumor sections. Moreover, sampling from a combination of edge and center areas in whole tumor sections would be ideal. A study in patients with primary NSCLC who underwent surgical resection revealed that three or four core biopsy specimens are the optimal minimum number for the determination of PD-L1 expression in whole tumor sections, which show a sensitivity value higher than 0.9 at cutoffs of 1 and 50% (60). According to another report, a minimum of 100 tumor cells in a single tumor tissue biopsy sample is required to evaluate PD-L1 expression for predicting the response of patients with nonsquamous NSCLC to nivolumab therapy (61).

**Figure 1:**
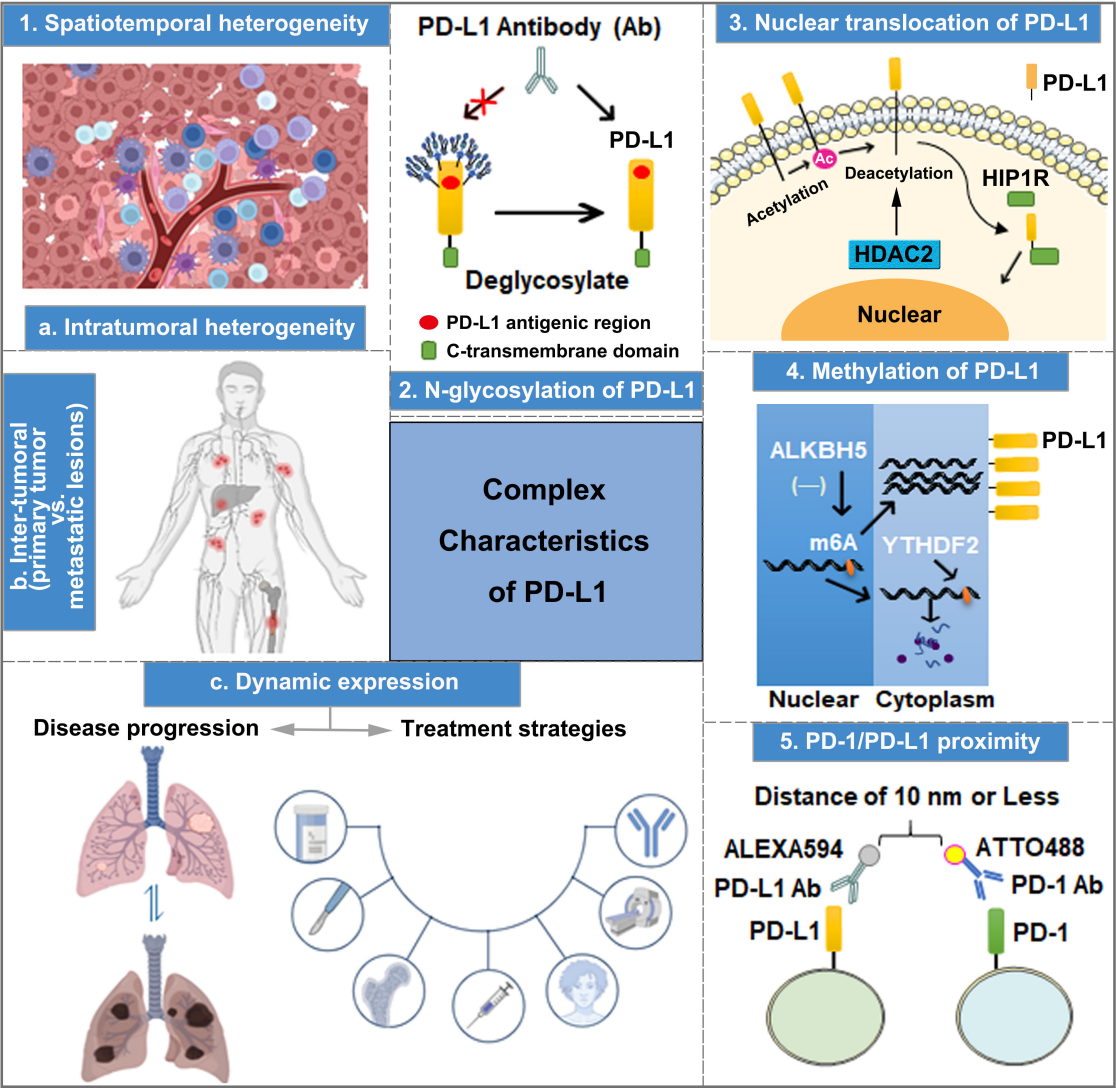
The complex characteristics of PD-L1 play important roles in predicting the efficacy of anti-PD-1/PD-L1 therapy, including spatiotemporal heterogeneity, transcriptional and post-translational modification, nuclear translocation, and PD-1 and PD-L1 interaction.

It is still difficult to overcome the temporal heterogeneity of PD-L1 expression in tumor tissues using IHC assays alone. Real-time detection of dynamic changes in PD-L1 expression may be an ideal means, so researchers use radioisotope labeling, a dynamic mapping and real-time quantitative analysis of PD-L1 expression to reduce the prediction error, which is due to the dynamic changes of PD-L1 expression during treatment. The molecular imaging of PD-L1 expression and dynamics with positron emission tomography (PET) or single-photon emission computed tomography (SPECT) using radiolabeled antibodies targeting PD-L1 has shown clinical applications (62–65). Hannan et al. developed a novel probe ^99m^Tc-MY1523 targeting PD-L1, which is conducive to the efficacy prediction of PD-L1 blockade immunotherapy (65). The probe comprises a nanobody MY1523, which has a high binding affinity and specificity to PD-L1, and ^99m^Tc radioisotope labeling. ^99m^Tc-MY1523 shows high tissue permeability and fast metabolism, revealing a high tumor contrast at 1 to 2 h post-injection. In mouse bearing A20, MC-38, and 4T1 tumor models, ^99m^Tc-MY1523 SPECT/CT allowed the dynamic mapping and real-time quantitative analysis of PD-L1 expression, which was upregulated by IFN-γ intervention. Hence, it considers the dynamic expression of PD-L1 in tumors and enhances PD-L1 blockade immunotherapy efficacy (**Figure 1**). Another clinical trial demonstrates that SPECT/CT assessment of PD-L1 expression in NSCLC patients can present and image characteristics correlating with PD-L1 immunohistochemistry well by using ^99m^Tc-NM-01-labeled anti-PD-L1 single-domain antibody, which is safe and has favorable biodistribution (66).

### N-glycosylation of PD-L1

PD-L1 is a type of highly glycosylated protein, which can be N-glycosylated at sites N35, N192, N200, and N219. Particularly, the glycosylation of N192, N200, and N219 is crucial in maintaining PD-L1 protein stability by inhibiting PD-L1 degradation through the GSK3β-mediated 26S proteasome pathway (67). Importantly, due to the high level of PD-L1 N-linked glycosylation, PD-L1 surface polypeptide antigens cannot be fully recognized by anti-PD-L1 antibodies during the IHC analysis of tumor tissue samples (67, 68). Lee et al. performed immunofluorescence detection using anti-PD-L1 antibody after treating A549 and BT-549 cells with PNGaseF (a recombinant glycosidase that removes N-linked glycosylation), revealing enhanced PD-L1 fluorescence intensity compared with the control group. Moreover, the binding affinity of PD-L1 antigen–antibody was increased by 25 and 55 times after PD-L1 expression in A549 and H1299 cells was modified by deglycosylation, respectively (68). Thus, exposing non-glycosylated PD-L1 antigens to the diagnostic antibodies of PD-L1 by removing the glycan structures on PD-L1 is a practical strategy for improving the accuracy of PD-L1 measurement (**Figure 1**), which aids in predicting the effectiveness of anti-PD-1/PD-L1 immunotherapy.

### Nuclear translocation of PD-L1

Apart from cell membrane surface distribution, PD-L1 can also undergo nuclear translocation (**Figure 1**), and nuclear PD-L1 (nPD-L1) expression is associated with a poor prognosis. Blocking PD-L1 nuclear translocation contributes to immune-related gene reprogramming and enhances anti-tumor response to PD-1 blockade (69). As a transcription factor, nPD-L1 promotes the expression of molecules associated with the immune response, which are not associated with PD-1/PD-L1 blockade targets after acetylation by EP300 acetyltransferase on the K263 residue. Moreover, the combination of PD-1/PD-L1 blockade and HDAC2 inhibitor reduces the acetylation-dependent PD-L1 nuclear localization and acquired immunotherapy resistance (70). Furthermore, PD-L1 nuclear translocation facilitates NSCLC cell proliferation through the Gas6/MerTK signaling pathway using KPNB1 as a cytoplasm-to-nucleus partner. nPD-L1 promotes Gas6 transcription by coupling with transcription factor Sp1, which results in Gas6 secretion and MerTK activation (71). Therefore, nPD-L1 measurement in combination with KPNB1 or MerTK detection could improve the accuracy of clinical anti-PD-1/PD-L1 treatment efficacy prediction. Additionally, another physiological role of PD-L1 nuclear translocation induced by hypoxia has been identified in MDA-MB-231 cells, wherein nPD-L1, in cooperation with p-STAT3, converts TNFα-induced apoptosis to non-canonical pyroptosis, which is mediated by the GSDMC/caspase-8 pathway, and leads to a poor antitumor immune response (72). Therefore, evaluating the efficacy of ICI treatment based on nPD-L1 detection through IHC in tumor tissues could be an effective strategy to improve the prediction accuracy.

### Methylation of PD-L1

DNA methylation has been reported to affect tumorigenesis. Moreover, it works as a biomarker for the diagnosis, response, and prognosis in cancer therapy (73, 74). Accordingly, Xue et al. developed an initial DNA methylation profile to predict the objective response rate (ORR) of PD-1/PD-L1 inhibition therapy and identify 269 CpG signatures related to ORRs in 18 cancer types (75). The hypermethylation of PD-L1 is associated with poor OS in several cancer types, such as colorectal cancer, prostate cancer, and melanoma. Pre-clinical evidence indicates that a combination of hypomethylating agents and ICIs could improve treatment efficacy (76). PD-L1 methylation (mPD-L1) can also be applied with other types of biomarkers to achieve increased prediction performance, which helps oncologists to select patients who are more likely to benefit from ICI therapy (77, 78). A study in intrahepatic cholangiocarcinoma indicates that a high ALKBH5 (m^6^A demethylase) expression in tumor cells inhibits m^6^A modification in the 3′-UTR region of PD-L1 mRNA, increasing the sensitivity to anti-PD-1 immunotherapy (**Figure 1**). Therefore, detecting mPD-L1 and ALKBH5 expression simultaneously could improve the accuracy of ICI treatment response prediction (79). A number of approaches for DNA methylation analysis have been used, such as direct Sanger sequencing, bisulfite sequencing PCR, pyrosequencing, methylation-specific PCR, and methylation-specific high-resolution melting. Moreover, the methylation of PD-L1 DNA can also be analyzed by using validated and registered kits for methylation detection (80).

### Assessments of PD-1/PD-L1 proximity

The interaction between PD-1 and PD-L1 has been reported to be responsible for immunosuppression. The recruitment of the tyrosine phosphatases (SHP-1 and SHP-2) to the immunoreceptor tyrosine-based switch motif of PD-1 induces the combination of PD-1 and PD-L1, thereby promoting an increase in immune evasion (81). After blocking the interaction between PD-1 and PD-L1 to inhibit the tumor immune escape, PD-L1 antibodies have better therapeutic efficiency (82–84). According to a study involving six laboratories, multiplex immunofluorescent (mIF) imaging approaches support PD-1/PD-L1 proximity assessments across multiple sites and contribute to the accurate quantification of %PD-L1 expression in NSCLC tissue sections (85). Compared to conventional single-IHC stains, six-plex mIF technology has multiple advantages, including high controllability, image analysis of multiple markers on a single or more slides, spatial relationship evaluation at the single-cell level, quantitative analysis of markers, inter-site comparison for PD-1/PD-L1 proximity, and implementation adapted to a daily schedule. However, the high operation requirements and reproducibility of staining intensity hinder its widespread clinical application. Therefore, a more rigorous and normalized operation protocol should be recognized and employed in prospective clinical trials and clinical practice. The emerging immune-Forster resonance energy transfer (iFRET) technology can be utilized to measure the distance between PD-L1/PD-1 on TCs or ICs, which reflects the degree of PD-1/PD-L1 interaction (86). The iFRET assay relies on two-site labeling that provides a quantitative read-out of protein–protein interactions between cells by fluorescence lifetime imaging microscopy, and FRET acts as a “chemical ruler”. First, PD-1 and PD-L1 are identified and labeled by their respective primary monoclonal antibodies. Then, two primary antibodies are stained with Fab fragments conjugated to the donor chromophore: ATTO488 for PD-1 and ALEXA594 for PD-L1 (**Figure 1**) separately. When the distance between PD-1 and PD-L1 is 1–10 nm, fluorescence change can be defined as positive (82). Clinical trials also show that PD-1/PD-L1 interaction is observed in patients with clear cell carcinoma of the kidney, including patients with negative PD-L1. Additionally, the degree of PD-1/PD-L1 interaction is positively correlated with the prognosis of patients with renal clear cell carcinoma, malignant melanoma, and metastatic NSCLC. Therefore, the detection of the interaction between PD-1 and PD-L1 using iFRET could be a potential approach in providing personalized immunotherapy.

## Liquid biopsy of PD-L1

**Figure 2:**
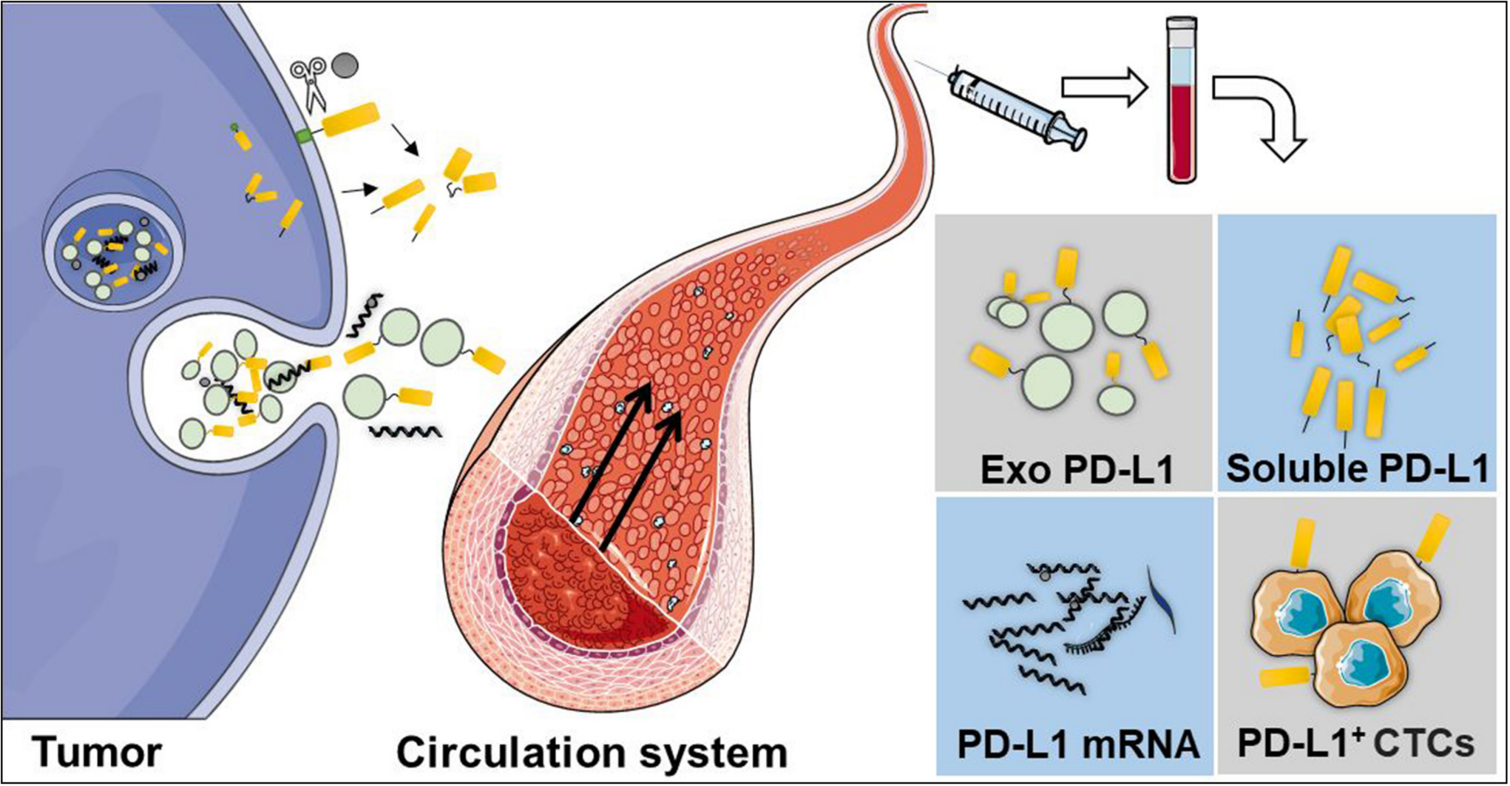
Liquid biopsy is a non-invasive method, which is safer and commonly preferred than traditional tissue biopsies. The dynamic changes of PD-L1 in the circulatory system can be detected using a liquid biopsy technique, including soluble PD-L1 (sPD-L1), exosomal PD-L1 (exoPD-L1), PD-L1 mRNA, and PD-L1 expression in circulating tumor cells.

Liquid biopsy is a non-invasive method which is safer and more commonly preferred than traditional tissue biopsies (87). It uses fluid samples from the circulatory system, including blood, cerebrospinal fluid, ascites, or other body fluids. Until now, liquid biopsy has been used for circulating tumor cells (CTCs), extracellular vesicles, circulating tumor DNA, and circulating free DNA detection (88). Moreover, liquid biopsy can be used to perform longitudinal analyses for monitoring therapy response (89, 90). Researchers have detected the dynamic changes of PD-L1 expression in the circulatory system by using a liquid biopsy technique, including soluble PD-L1 (sPD-L1), exosomal PD-L1 (exoPD-L1), blood PD-L1 mRNA, and PD-L1 expression in circulating tumor cells (PD-L1^+^CTCs) (**Figure 2**).

### sPD-L1

sPD-L1 can be translated from the splice variant mRNA lacking the transmembrane domain of PD-L1 (91) or cleaved from membrane-type PD-L1 using matrix metalloproteinases (92). In four patients experiencing a recurrence of NSCLC after anti-PD-L1 treatment, Gong et al. identified two types of stably expressed sPD-L1 (PD-L1v242 and PD-L1v229), which act as negative regulators in anti-PD-L1 antibody treatment by competing with membrane-type PD-L1 (93). After the inoculation of mice with MC38 mouse colon cancer cells that expressed sPD-L1 and membrane-type PD-L1 at a cell number of 1:99 followed by anti-PD-L1 antibody treatment, the whole tumor showed resistance to PD-L1 treatment despite 1% of tumor cells expressing sPD-L1. Additionally, Zhou et al. propose that the increase of sPD-L1 is negatively correlated with the prognosis of patients with renal cell carcinoma and multiple myeloma who received anti-PD therapy (91). However, the use of sPD-L1 in predicting the efficacy of ICIs at an early stage or before treatment remains unclear and requires further exploration.

### ExoPD-L1

ExoPD-L1 is present on the surface of tumor-derived exosomes and positively correlated with the PD-L1 expression on the cell surface, which can mediate immune escape in tumor tissues (69, 94). A high level of exoPD-L1 has been considered as one of the immunosuppressive factors that inhibit T cell activation and lead to ICI treatment resistance (95). After evaluating the PD-L1 expression in various tumor tissues, Poggio et al. suggested that cancer cells secret a large amount of PD-L1 in exosomes, while only a small part exists on the cell surface. In PD-L1 antibody-resistant tumor tissues, cancer cell growth is inhibited by removing exoPD-L1 by knocking out *RAB27A* (member RAS oncogene family) and *NSMASE2* (sphingomyelin phosphodiesterase 3) (96). Consequently, blocking exoPD-L1 is expected to remove the resistance to anti-PD-1/PD-L1 therapy. However, drugs that can effectively block exoPD-L1 are scarce. Currently, although GW4869 and Nexinhib-20 are drugs targeting key enzymes NSMASE2 and RAB27A in the exosome, restraining exosomal releases in various cancer cell lines, such as PC3 and MC38, proves difficult (97–99). Therefore, an effective drug that can block the release of exoPD-L1 and be combined with anti-PD-L1 antibody in cancer treatment could aid in achieving a better anti-tumor therapeutic effect (94).

Besides ELISA or the magnetic beads coupled with exoPD-L1 surface markers (such as CD63, CD9, CD81, et al.) that can be used to separate exosomes from the patient’s serum or plasma and determine the level of exoPD-L1 (100), a novel method for exoPD-L1 detection called HOLMES-Exo_PD-L1_ has been recently developed. A short single-stranded DNA (MJ5C) smaller than the PD-L1 antibody acts as an adapter of PD-L1. MJ5C is highly selective and can overcome the steric hindrance caused by PD-L1 glycosylation. Compared with the PD-L1 antibody, MJ5C shows higher molecular recognition ability. Additionally, thermal electrophoresis technology is utilized to bind PD-L1 in a homogeneous solution without separation. Compared with ELISA, HOLMES-Exo_PD-L1_ can promote the binding kinetics between aptamer and exoPD-L1 with a higher combination and detection efficiency (101).

### Blood PD-L1 mRNA

Yang et al. collected paired tissue and blood samples from 51 patients with advanced NSCLC after 2 months of ICI treatment to detect the expression of blood PD-L1 for correlation analyses. The positive tissue PD-L1 (tPD-L1) showed a significantly higher PD-L1 mRNA than those with negative tPD-L1. Moreover, the combination of PD-L1 mRNA and exoPD-L1 could be used to screen for patients suitable for immune checkpoint PD-1/PD-L1 blockades (102). However, whether the dynamic change of blood PD-L1 mRNA can serve as an optimistic biomarker in predicting immunotherapy efficacy requires further study.

### PD-L1 expression on CTCs

CTCs originate from the primary tumor and are distributed in the circulatory system as individuals or clusters, playing an important role in tumor metastasis and revealing tumor heterogeneity better than tissue biopsies (87, 88) CTC samples can be collected using liquid biopsy technique, and multi-time sampling can be carried out for longitudinal monitoring due to its non-invasive characteristic (103, 104). Research in NSCLC indicates that the analysis of PD-L1 expression on CTCs (PD-L1^+^CTCs) is a potential factor in overcoming the tumor biopsy spatiotemporal heterogeneity of PD-L1 expression (103). Similarly, Ilie et al. evaluate the levels of PD-L1 in CTCs and WBCs (white blood cells) and point out the potential of CTC assessment as a real-time biopsy to detect PD-L1 expression in patients with NSCLC (105). At present, the CellSearch system is the only method approved by the FDA for CTC detection (88). Sinoquet et al. report that PD-L1^+^CTCs can predict clinical therapeutic effects in patients with NSCLC using the FDA-cleared CellSearch^®^ analysis system. A study in head and neck cancer patients also highlights that PD-L1^+^CTCs are positively correlated with response to anti-PD-L1 therapy (106).

However, contrary reports do exist (107–109)—for example, in patients with advanced NSCLC, Guibert et al. found that PD-L1^+^CTC analysis is highly feasible, and CTCs were more PD-L1-positive than in tissues. However, there is no correlation between PD-L1 expression in tissues and on CTCs. Moreover, PD-L1 expression on CTCs has no remarkable prognostic impact (107). The scarcity of CTCs in the circulatory system and the lack of unified guidelines for clinical PD-L1^+^CTC analysis could be responsible for the poor accuracy and inconsistent results (110). Perhaps the effective enrichment of CTCs can be an effective strategy for improving accuracy. Additionally, the validity and uniformity of PD-L1^+^CTC test results require a large-scale clinical validation.

## The combination of PD-L1 and potential tumor type-dependent biomarkers

A positive or negative regulatory relationship exists between specific markers and PD-L1 in different cancers. Herein we propose that PD-L1 combined with specific markers of different tumor cells might improve prediction accuracy.

In K-RAS-driven pancreatic cancer specimens, PD-L1 is highly expressed, which is induced by exogenous ROS and FGFR1 signal activation. Antioxidants and FGFR1 knockout cause a decrease in PD-L1 expression and a remarkable increase in T cell-mediated tumor suppression (111). Research shows that CDKN2A is significantly upregulated in PD-L1 blockade therapy responders (112). The expression of tumor cell-intrinsic PD-L1 can be increased *via* RAS-MEK signaling, which modulates PD-L1 mRNA stability and strengthens immune escape in cancer (113). Perhaps RAS oncogenes can be used as a joint marker to predict efficacy in combination with PD-L1 in patients treated with ICIs. Moreover, in patients with non-squamous NSCLC, the functional STK11 mutations result in resistance to PD-1/PD-L1 blockade (anti-PD-L1 antibody durvalumab ± anti-CTLA-4 antibody tremelimumab) immunotherapies, highlighting STK11 as a potential co-biomarker in screening the optimal subpopulation for personalized immune checkpoint therapy (114). A study in hepatocellular carcinoma indicates that, apart from the CPS of PD-L1, Wnt/β-catenin activation and CD8^+^ tumor-infiltrating lymphocyte (TIL) numbers are also conducive in predicting the response to anti-PD-1 antibody treatment (115). The positive expression of human endogenous retrovirus-H long terminal repeat-associating protein 2 (HHLA2), a new member of the B7 family, has been associated with a significantly shorter OS and progression-free survival (PFS) in clear cell renal cell carcinoma (ccRCC). When compared with HHLA2^−^/PD-L1^−^, HHLA2^−^/PD-L1^−^, and HHLA2^−^/PD-L1^+^ groups, HHLA2^+^/PD-L1^+^ shows the highest density of CD8^+^ and CD4^+^ TILs and risk of ccRCC progression. Therefore, HHLA2^+^/PD-L1^+^ is positively associated with poor response to ICI treatment in patients with ccRCC (116).

Briefly, there exists a close correlation between non-immune signals and immune checkpoint blockade outcomes. Perhaps a combination of PD-L1 and specific molecular biomarkers could be suitable for the screening of subpopulations that show a good response to ICI therapies.

## The expression of PD-L1 on different subpopulations of non-tumor cells

Except for tumor cells, PD-L1 is also widely expressed on various immune and non-immune cells, such as T cells, macrophages, B cells, dendritic cells, neutrophils, and fibroblasts. PD-L1 expression on non-tumor cells participates in antitumor immunity regulation by interacting with tumor cells or other cells to affect the efficacy of anti-PD-1/PD-L1 therapy in patients (117).

### T cells

After T cell activation, immune-inhibitory receptor PD-1 is expressed by stimulated CD4^+^ and CD8^+^ T cells and restrains antitumor immune responses (118). In a pancreatic ductal adenocarcinoma mouse model, Diskin et al. demonstrate that PD-L1 is expressed on ~40% of CD4^+^ T cells and ~60% of CD8^+^ T cells (28). The number of PD-L1^+^ T cells increases with tumor progression; thus, PD-L1 expression on T cells might play a vital role in forecasting the therapeutic effect of anti-PD-L1 antibodies. In NSCLC, Wu et al. find that patients with a high level of PD-L1^+^CD25^+^CD4^+^ T cell (Treg cell) abundance have a better response to ICI treatments (27).

### Macrophages

It has been reported that PD-L1 expression on macrophages promotes tumor resistance to anti-PD-1 antibody by interacting with CD80 expressed on T cells and inducing their differentiation into regulatory T cells, thereby causing tumor resistance to anti-PD-1 antibody (29). However, studies also show that PD-L1 expression on macrophages may have no impact on anti-PD therapy—for example, in the early stage of lung cancer, PD-L1 expression on most tumor-associated macrophages (TAM) does not affect the tumor cell killing by tumor-specific T cells (30), which only plays a role in regulating the interaction between TAM and homologous effector T cells and protecting TAM from being killed by T cells. This part of PD-L1 does not participate in the process of T cells killing tumor cells, even if, after treatment with anti-PD antibody, patients still do not achieve a remarkable therapeutic effect. Therefore, the anti-tumor immune response of PD-L1 expression on macrophages is different based on the tumor type and tumor development stage, so it is still controversial whether macrophage-expressed PD-L1 can be used as a biomarker to predict the efficacy of ICI therapy.

### Dendritic cells

Dendritic cells (DCs) represent a significant source of PD-L1. DCs in tumor tissues are known to play critical roles in tumor antigen cross-presentation and activating CD8^+^ T cells in the lymph nodes (119), which mediate the initiation of immune response and regulate the function of multiple immune cells (120). CD80 expressed on DCs can bind to CD28 on T cells to activate T cells. Specifically, on the one hand, PD-L1 expression on DCs directly inhibits T cell activation by binding to PD-1 on T cells. On the other hand, it prevents CD80 from interacting with CD28 on T cells by competitively binding to CD80 and then inhibits T cell activation indirectly (121). After treatment with anti-PD-L1 antibody, the signal intensity of CD80 and CD28 binding is increased from 32 to 52% based on the results of Tag-lite detection technology, and DCs initiate T cell activation and proliferation when PD-L1 is blocked. Furthermore, patients with high DC abundance are found to have 75% lower risk of death compared to those with low DC abundance. Lin et al. also prove that PD-L1 expressed on DCs is valuable for predicting the therapeutic efficacy of immune checkpoint blockades in melanoma and ovarian cancer treatment (122). Therefore, PD-L1 expression on DCs might become the target of PD-L1 antibody (33, 123). Even PD-L1 expression on CD11c^+^ DCs show potential in predicting the efficacy of anti-PD therapy, but not all DCs can express PD-L1. Hence, after treating the tumor cells with CD11c and anti-PD-L1 antibodies, immunofluorescence analysis can be performed by flow cytometry or laser scanning confocal microscopy to quantitatively determine the level of PD-L1 expression on CD11c^+^ DCs. Despite that the cost might be higher than IHC testing of total PD-L1 in tumor tissues, the prediction results of immunofluorescence analysis can be more accurate.

### Other non-tumor cells

In addition to the non-tumor cells mentioned above, PD-L1 expression on neutrophils, fibroblasts, and B cells also has been reported to play an important role in regulating anti-tumor immunity and influencing patients’ response to anti-PD therapy (124). Shi et al. discover that gastric cancer cell-derived extracellular vesicles can induce PD-L1 expression on neutrophils through the STAT3 pathway, and PD-L1^+^ neutrophils can suppress the function of T cells and facilitate gastric cancer progression *via* promoting the PD-1/PD-L1 interaction (34). Research in cancer-associated fibroblasts of NSCLC patients suggests that IFN-γ induces anti-tumor immune responses and contributes to a better prognosis of patients by upregulating the expression of PD-L1 (35). Adnan et al. find that PD-L1 expression on regulatory B cells can weaken the humoral immune response mediated by follicular helper T cells (31). Moreover, the continuous activation of IL-21/STAT3/IRF1 and CD40L/ERK signals that induced the PD-L1 upregulation on B cells inhibits CD8^+^ T cell function (32).

## The combination of PD-L1 with the features of tumor microenvironment

The complex tumor microenvironment (TME) is one of the key factors that affect tumor immunotherapy response (125). The 3D organoid system for the culture of different cell groups in a three-dimensional environment is more similar to the *in vivo* microenvironment and can retain the heterogeneity originality of the tumor tissues (126). Patient-derived *ex vivo* organoid models for the dynamic monitoring of PD-L1 expression have been reported. *Ex vivo* assays for immunotherapy response evaluation in patients guarantee that all tumor and non-tumor cells of the TME can survive, with testing lasting for at least 7–10 days to capture the effects of immune therapies (126).

To assess the early response of different cancer types in *ex vivo* PD-1 blockade, a patient-derived tumor fragment (PDTF) platform based on the 3D organoid culture has been developed (127). In this system, fresh surgical tumor tissues are fragmented to approximately 1 mm^3^ to allow sufficient nutrient and reagent intake while preserving the cellular architecture of the tumor. The fragments are then embedded into the artificial extracellular matrix. During culturing, cellular and soluble factors are monitored continuously at different timepoints after anti-PD-1 treatment. To determine the correlation between the *ex vivo* immunological response of surgical lesions and clinical response, 12 patients subsequently treated with PD-1 blockade were selected. Furthermore, the *ex vivo* PDTF outcomes of the 12 cases are fully consistent with the clinical response. Moreover, based on the phenotype analysis of T cells in PDTF responder tumors, PD-1^+^CD8^+^ T cells (CD8^+^ T cells with a higher PD-1 expression level than that in peripheral blood T cells), PD-1^T^CD4^+^ T cells, and PD-1^T^CD45^+^ lymphocytes were increased, which strongly reflect the anti-PD-1 response given the high area under the curve ≥0.84. However, based on previous studies, this technique can support multiple factors to efficiently predict and screen for optimal subpopulations rather than the use of a single factor like PD-L1 expression (128). Therefore, to a certain degree, patient-derived *ex vivo* organoid technology furthers the application of personalized and precise ICI therapy and the development of more sensitive biomarkers for predicting the response in pre-clinical and clinical trials.

## Spatially informed simultaneous evaluation of multiple biomarkers: digital spatial profiling technology

Actually, the spatio-temporal heterogeneity of PD-L1 expression in tumor tissues, the PD-L1 expression on various immune cells or fibroblasts, and the complex tumor microenvironment are important factors leading to the many limitations of one-dimensional IHC analysis in PD-L1 detections. Therefore, multi-dimensional space needs to be considered. Maria et al. proved that digital spatial profiling (DSP) technology using a 44-plex antibody cocktail could be used to find potential novel biomarkers for the prediction of immunotherapy response in melanoma. It was demonstrated that high CD3, CD8, CD11c, HLADR, IDO1, and TIM3 in tumor were predictive for PFS. In macrophages, high CD3, CD4, CD8, PD-L1, and beta-2-microglobulin (B2M) were associated with a longer overall survival. High B2M was predictive in lymphocytes (129). High throughput and automation are the advantages that belong to DSP technology, but the cost is high. Although DSP has been used to identify novel biomarkers for predicting response to ICI therapies in different tumor types, including NSCLC (130) and melanoma (83), prior to its clinical translation, a standardized instruction manual for increasing operator convenience and obtaining a more precise analysis is still needed (131).

## Conclusions

Various clinical studies have adopted PD-L1 to screen for patients suitable for ICI treatment and formulate personalized immunotherapy regimes, which can reduce the psychological, physical, and economic burden of the patients. This review summarizes the challenges of PD-L1 expression as a predictive biomarker for anti-tumor efficacy prediction in anti-PD-1/PD-L1 immunotherapy. Furthermore, the feasibility of the current PD-L1 expression detection and their practical improvement methods are proposed.

Currently, numerous pre-clinical and clinical research data and novel detection technologies are indispensable to precisely assess PD-L1 expression. However, international standardized protocols regarding clinical sample acquisition and processing, antibody clone number selection, and standardized data analyses are required, which are curated to specific detection platforms. Moreover, for different cancer types, the development of more sensitive and combined biomarkers related to PD-L1 expression is a potential avenue to explore. Finally, compared with tumor tissue samples, liquid biopsy has great benefits, especially in reducing the patient’s pain and in real-time PD-L1 detection in the circulatory system. In **Table 2**, we preliminarily evaluated the usefulness of different PD-L1 detection methods mentioned above in daily implementation, including the cost, transformation time, and accreditation, which was done for reference.

**Table 2:** Preliminary evaluation of the usefulness of different PD-L1 detection techniques.

Optimization of indicators and strategies			Cost	Turnaround time	Accreditation	References
PD-L1 in tumor tissues	Intra/inter-tumoral heterogeneity of PD-L1	Immunohistochemistry (IHC): a. Core number: three or four b. Cell numbers of each core: ≥100 c. The size of biopsy is not less than 8 mm^2^ d. Detection of PD-L1 in the primary and metastatic tumor lesions is necessary	+	+	+++	(44, 48, 59, 60)
	Dynamic expression of PD-L1	SPECT/PET (99mTc-MY1523- or 99mTc-NM-01-labeled)	+	+	++	(61–65)
	N-glycosylation of PD-L1	PNGaseF-IHC	+	++	+	(67)
	Nuclear translocation of PD-L1	IHC	+	+	++	(68–71)
	Methylation of PD-L1	BSP/MS-PCR/MS-HRM/registered kits	++	++	+	(74–79)
	Assessments of PD-1/PD-L1 proximity	Six-plex mIF technology	+++	++	+	(84)
		iFRET technology	++	+	+	(85)
Liquid biopsy of PD-L1	sPD-L1	ELISA	+	+	++	(90–92)
	ExoPD-L1	ELISA, HOLMES-ExoPD-L1 quantitation method, or Simoa TM PD-L1 Kit	+	+	+	(93–100)
	Blood PD-L1 mRNA	RT-QPCR	+	+	+	(101)
	PD-L1^+^CTCs	CellSearch^®^ analysis system	+	+	+++	(87, 102–105)
PD-L1 expression on non-tumor cells	Immune cells	Multi-dimensional: digital spatial profiling technology	+++	++	++	(129–131)
	Fibroblasts					

The symbol “+” represents the degree of accreditation: *in vivo*+—cell, *in vivo*, and retrospective studies; *in vivo*++—cell, *in vivo*, retrospective studies, and prospective clinical trials; +++—Food and Drug Administration and European Medicines Agency approval.

So far, in addition to PD-L1, various biomarkers have been proved to be related to a better response rate in anti-PD-1/PD-L1 therapies, including tumor mutation burden, high microsatellite instability, neutrophil-to-lymphocyte ratio, deficient mismatch repair, TILs, tumor inflammation signature (TIS), T cell CX3C chemokine receptor 1 (CX3CR1) expression, *etc.* (132–137). Therefore, the combined detections of the above-mentioned indicators can also be considered candidate strategies in comprehensively determining the prognosis of ICI therapies.

**Figure 3:**
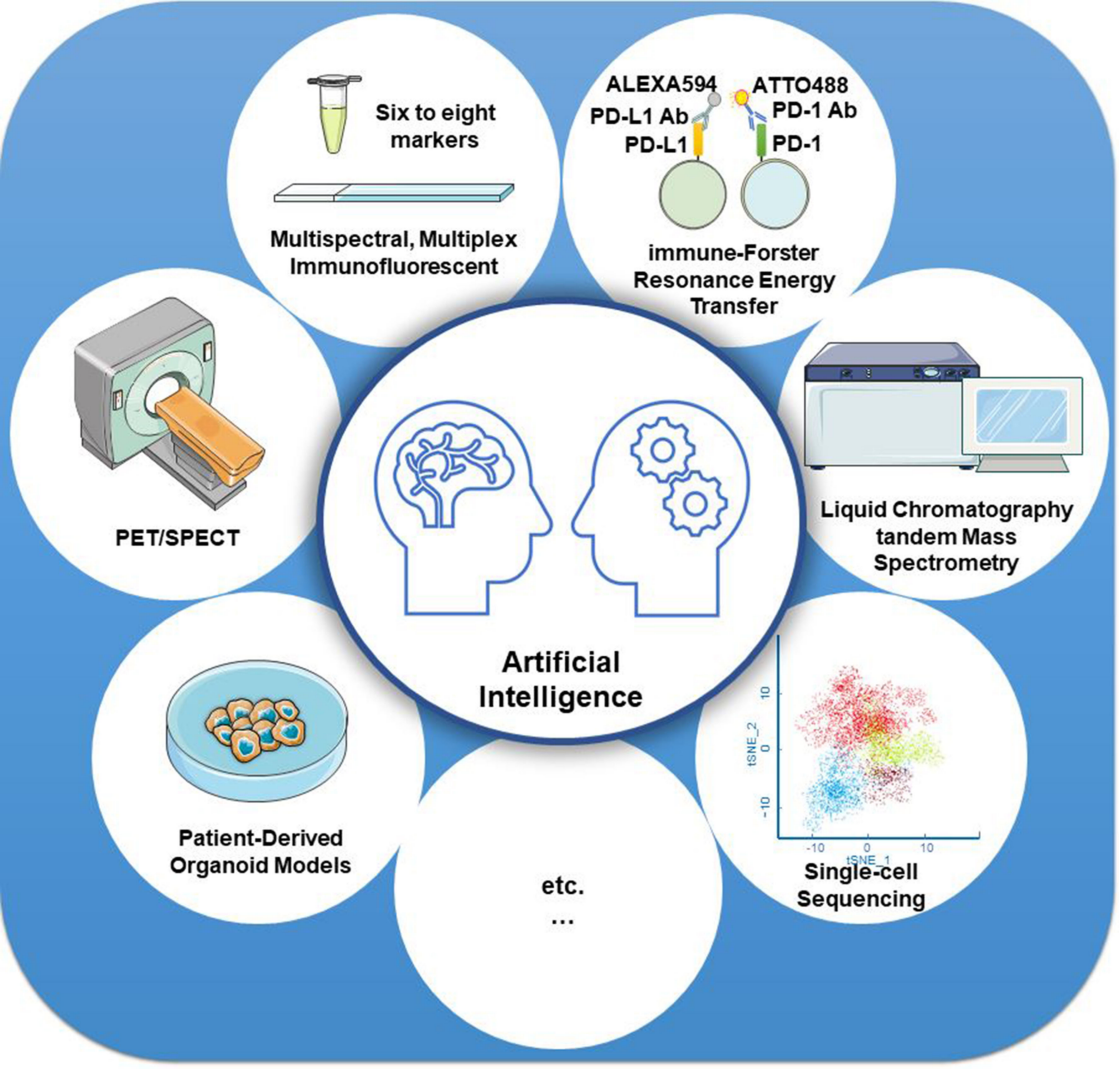
Strategies for improving the detection accuracy of PD-L1. Artificial intelligence systems in combination with other technologies, such as mIF imaging, iFRET assay, PET or SPECT, liquid chromatography tandem mass spectrometry, patient-derived *ex vivo* organoid models, and single-cell sequencing, can revolutionize the clinical application of PD-L1 evaluation, especially in predicting the efficacy of PD-1/PD-L1 blockades.

Furthermore, new technologies, such as artificial intelligence (AI), have also been used in the precise assessment of PD-L1 detection. Based on the AI system, the computer-assisted PD-L1 score is highly consistent with the pathologists’ score, thereby improving test reproducibility and providing a promising diagnostic tool in clinical pathology (138–140). One recent AI-assisted diagnosis study confirmed that the AI system contributed to enhancing the efficiency and repeatability of untrained pathologists’ operations. TPS calculations of PD-L1 expression in NSCLC indicated a high consistency between the AI system and the pathologists (*R* = 0.9787) based on the Ventana PD-L1 (SP263) assay (141). AI systems in combination with other technologies, such as mIF imaging, iFRET assay, PET or SPECT, liquid chromatography tandem mass spectrometry, patient-derived *ex vivo* organoid models, and single-cell sequencing (**Figure 3**), can revolutionize the clinical application of PD-L1 evaluation, especially in predicting the efficacy of PD-1/PD-L1 blockades.

## Author contributions

DL, JZ, and MS: conceptualization, supervision, investigation, funding acquisition, and writing—review and editing. XZ: writing—original draft, writing—review and editing, and funding acquisition. YB, BM, ZX, SL, XW, RH, and WM: investigation, visualization, and writing—review and editing. All authors contributed to the article and approved the submitted version.

## Funding

This work was supported by the National Natural Science Foundation of China (no. 82003164 and 81972719), Jiangsu Province Natural Science Foundation (no. BK20201012), Science and Technology Project of Xuzhou (no. KC20126), National Science Research in Universities of Jiangsu Province (no. 20KJB320032 and 21KJA320008), Key Research and Development Plan of Xuzhou (no. KC18102), and Scientific Research Foundation of Xuzhou Medical University (no. D2019050).

## Conflict of interest

The authors declare that the research was conducted in the absence of any commercial or financial relationships that could be construed as a potential conflict of interest.

## Publisher’s note

All claims expressed in this article are solely those of the authors and do not necessarily represent those of their affiliated organizations, or those of the publisher, the editors and the reviewers. Any product that may be evaluated in this article, or claim that may be made by its manufacturer, is not guaranteed or endorsed by the publisher.

## Glossary

**Table:**

PD-1	programmed cell death-1
PD-L1	programmed cell death ligand 1
ICIs	immune checkpoint inhibitors
IHC	immunohistochemistry
NSCLC	non-small cell lung cancer
OS	overall survival
TNBC	triple-negative breast cancer
ICs	immune cells
TPS	tumor proportion score
EGFR	epidermal growth factor receptor
PET	positron emission tomography
SPECT	single-photon emission computed tomography
RCC	renal cell carcinoma
nPD-L1	nuclear PD-L1
ORRs	objective response rates
mPD-L1	PD-L1 methylation
HDAC2	histone deacetylase 2
Gas6	growth arrest-specific 6
MerTK	MER proto-oncogene tyrosine kinase
KPNB1	karyopherin β1
TNF-α	tumor necrosis factor-α
STAT3	signal transducers and activators of transcription 3
GSDMC	gasdermin C
SHP-1	Src homology region 2 domain-containing phosphatase-1
SHP-2	Src homology region 2 domain-containing phosphatase-2
mIF	multiplex immunofluorescent
iFRET	immune-Forster resonance energy transfer
CPS	combined positive score
ROS	reactive oxygen species
FGFR1	fibroblast growth factor receptor 1
CDKN2A	cyclin-dependent kinase inhibitor 2A
UC	urothelial carcinoma
RCC	renal cell carcinoma
RAS	rat sarcoma
MEK	mitogen-activated protein kinase kinase 7
MAP	mitogen-activated protein
ERK	extracellular regulated MAP kinase
HCC	hepatocellular carcinoma
HNSCC	head and neck squamous cell carcinoma
HHLA2	human endogenous retrovirus-h long terminal repeat-associating protein 2
TILs	tumor-infiltrating lymphocytes
PFS	progression-free survival
ccRCC	clear cell renal cell carcinoma
CTCs	circulating tumor cells
sPD-L1	soluble PD-L1
exoPD-L1	exosomal PD-L1
RAB27A; NSMASE2;	member RAS oncogene family; sphingomyelin phosphodiesterase 3;
HOLMESE_xoPD-L1_	homogeneous low-volume, efficient, and; sensitive exosomal PD-L1
tPD-L1	tissue PD-L1
FDA	United States Food and Drug Administration
IFN-γ	interferon gamma
LDT	laboratory development test
TME	tumor microenvironment
PDTF	patient-derived tumor fragment
DSP	digital spatial profiling
IDO1	indoleamine 2,3-dioxygenase 1
B2M	beta-2-microglobulin
TMB	tumor mutation burden
MSI-H	high microsatellite instability
NLR	neutrophil-to-lymphocyte ratio
dMMR	deficient mismatch repair
TIS	tumor inflammation signature
CX3CR1	cx3c chemokine receptor 1
AI	artificial intelligence
GEJ	gastroesophageal junction
ESCC	esophageal squamous cell carcinoma
